# Effect of the Mediterranean diet on the faecal long-chain fatty acid composition and intestinal barrier integrity: an exploratory analysis of the randomised controlled LIBRE trial

**DOI:** 10.1017/S0007114524001788

**Published:** 2024-11-14

**Authors:** Benjamin Seethaler, Maryam Basrai, Audrey M. Neyrinck, Walter Vetter, Nathalie M. Delzenne, Marion Kiechle, Stephan C. Bischoff

**Affiliations:** 1 Institute of Nutritional Medicine, University of Hohenheim, Stuttgart, Germany; 2 Metabolism and Nutrition Research Group, Louvain Drug Research Institute, UCLouvain, Université Catholique de Louvain, Brussels, Belgium; 3 Institute of Food Chemistry, University of Hohenheim, Stuttgart, Germany; 4 Department of Gynecology, Center for Hereditary Breast and Ovarian Cancer, Klinikum Rechts der Isar, Technical University Munich and Comprehensive Cancer Center Munich, Munich, Germany

**Keywords:** Mediterranean diet, Faecal fatty acids, Long-chain fatty acid, Gut barrier, Intestinal barrier, Gut permeability

## Abstract

We recently showed that adherence to the Mediterranean diet increased the proportion of plasma *n*-3 PUFA, which was associated with an improved intestinal barrier integrity. In the present exploratory analysis, we assessed faecal fatty acids in the same cohort, aiming to investigate possible associations with intestinal barrier integrity. Women from the Lifestyle Intervention Study in Women with Hereditary Breast and Ovarian Cancer (LIBRE) randomised controlled trial, characterised by an impaired intestinal barrier integrity, followed either a Mediterranean diet (intervention group, *n* 33) or a standard diet (control group, *n* 35). At baseline (BL), month 3 (V1) and month 12 (V2), plasma lipopolysaccharide-binding protein, faecal zonulin and faecal fatty acids were measured. In the intervention group, faecal proportions of palmitoleic acid (16:1, *n*-7) and arachidonic acid (20:4, *n*-6) decreased, while the proportion of linoleic acid (18:2, *n*-6) and *α* linoleic acid (18:3, *n*-3) increased (BL-V1 and BL-V2, all *P* < 0·08). In the control group, faecal proportions of palmitic acid and arachidic acid increased, while the proportion of linoleic acid decreased (BL-V1, all *P* < 0·05). The decrease in the proportion of palmitoleic acid correlated with the decrease in plasma lipopolysaccharide-binding protein (ΔV1-BL *r* = 0·72, *P* < 0·001; ΔV2-BL *r* = 0·39, *P* < 0·05) and correlated inversely with adherence to the Mediterranean diet (Mediterranean diet score; ΔV1-BL *r* = –0·42, *P* = 0·03; ΔV2-BL *r* = -0·53, *P* = 0·005) in the intervention group. Our data show that adherence to the Mediterranean diet induces distinct changes in the faecal fatty acid composition. Furthermore, our data indicate that the faecal proportion of palmitoleic acid, but not faecal *n*-3 PUFA, is associated with intestinal barrier integrity in the intervention group.

The intestinal barrier protects the host against gut microbes, food antigens and toxins present in the gastrointestinal tract. A functioning intestinal barrier is required for gut health, whereas intestinal barrier impairment is associated with a wide range of noncommunicable diseases, including CVD, cancer, type 2 diabetes and inflammatory bowel disease^(1,2)^.

Intestinal barrier integrity is affected by several endogenous and exogenous factors including diet, stress, excessive body weight and low or extreme physical activity^(1,3,4)^. To assess intestinal barrier integrity in larger cohorts, we have recently validated plasma lipopolysaccharide-binding protein (LBP) and faecal zonulin as suitable biomarkers^(5)^.

Even though it has been shown that the intestinal barrier plays a central role in health and disease, and the interest in this topic is increasing rapidly, the mechanisms by which the barrier function is regulated are not well known. We and others have shown that SCFA, derived from bacterial fermentation of dietary fibres, improve intestinal barrier integrity^(6–9)^. Also, vitamins, minerals, amino acids and polyphenols might have an effect^(10,11)^. For the first time in a human trial, we have recently shown that diet-derived *n*-3 PUFA, originated from increased adherence to the Mediterranean diet and assessed in plasma, are associated with an improvement in a previously impaired intestinal barrier integrity^(12)^. Data for this study were derived from the randomised controlled LIBRE (Lifestyle Intervention Study in Women with Hereditary Breast and Ovarian Cancer) trial, which included women at high risk for breast and ovarian cancer due to a pathogenic germline mutation in the BRCA1 and/or BRCA2 genes^(13)^. These mutations have been shown to be associated with an altered intestinal barrier integrity^(6,14)^. In detail, LIBRE study participants showed a higher median concentration of faecal zonulin at baseline (BL) compared with a healthy cohort of thirty-six women with a similar range of age and BMI (178 ng/mg *v*. 110 ng/mg, *P* < 0·01, Mann–Whitney *U* test)^(5)^. It has been suggested that intestinal barrier impairment may be linked to breast cancer initiation and progression^(15)^, which is the reason why we are interested in investigating factors that alter intestinal barrier integrity.

We have previously shown that SCFA induced by dietary fibres from the Mediterranean diet stabilise intestinal barrier integrity^(6)^. Now we wondered if long-chain fatty acids (LCFA) also regulate intestinal barrier function. We previously found that the proportion of plasma *n*-3 PUFA, as well as plasma saturated fatty acids, was associated with an improvement in intestinal barrier integrity^(12)^. Also, recent studies revealed that fibre supplementation alters the faecal proportions of LCFA^(16,17)^. Since these previous findings are of clinical relevance, we have decided to investigate these in more detail in the LIBRE study. The aim of the present study is to investigate possible associations between the fibre-rich Mediterranean diet^(18)^, faecal proportions of LCFA and biomarkers of intestinal barrier integrity.

## Materials and methods

### Study design

Data for the present exploratory study were derived from the randomised controlled LIBRE 1 trial^(13)^. This study was a randomised (1:1 ratio; group allocation performed centrally using randomly permuted blocks of length 2–6; participating centre and previous breast cancer were used as stratification factors), prospective, open-label, two-armed controlled multicentre trial, aiming to test the effect of a structured lifestyle intervention programme focusing on the Mediterranean diet and increased physical activity on cancer-relevant outcomes. This study started in 2014 and included women at high risk for breast and ovarian cancer due to a pathogenic germline mutation in the BRCA1 and/or BRCA2 genes.

The primary endpoint was the number of participants who successfully completed the first 3 months of lifestyle intervention. A rate of 70 % adherence or more was considered a success. Secondary endpoints comprised BMI, physical fitness and the analyses of omega fatty acids and faecal metabolites. The sample size in LIBRE-1 was adjusted to this goal but was not calculated based on statistical assumptions and tests, as described in the study protocol^(13)^. The LIBRE-2 confirmatory study, which aims to include 600 women, started in 2015, and recruitment is ongoing^(19)^.

In LIBRE, women with a history of breast cancer prior to the study start as well as women without previous breast cancer were included. Inclusion criteria were female sex, age between 18 and 69 years, a pathogenic BRCA1/2 mutation and written informed consent. Exclusion criteria comprised, among others, a BMI below 15 kg/m^2^ and neoplastic diseases currently in treatment^(13)^.

Individuals from the intervention group (*n* 33) received a structured lifestyle intervention programme, consisting of a 3-month intensive phase with biweekly group classes on the Mediterranean diet as well as professionally guided sports training. The intensive phase was followed by a 9-month less intensive phase with monthly meetings. The control group (*n* 35) was lectured once on the dietary recommendations of the German Nutrition Society (DGE) and once on the beneficial effects of regular physical activity on breast cancer incidence, prognosis and recurrence at the beginning of the study. Study visits were at BL, as well as 3 months (time point V1) and 12 months (time point V2) after BL. Details on the enrolment, randomisation, drop-outs and available data for each time point are shown in the CONSORT flow chart in online Supplementary Fig. 1.

This study was conducted according to the guidelines laid down in the Declaration of Helsinki, and all procedures involving human subjects were approved by the ethics review board of the Klinikum Rechts der Isar of the Technical University of Munich (reference no. 5686/13). Written informed consent was obtained from all subjects. The trial was registered at clinicaltrials.gov (reference no. NCT02087592).

### Dietary measurements

Two validated dietary questionnaires were used. The Mediterranean Diet Adherence Screener (MEDAS) was developed in the Prevención con Dieta Mediterránea (PREDIMED) studies^(20,21)^. We calculated the MEDAS-Score as the percentage of the achieved score related to the achievable score (e.g. 7/14 = 50 %; 7/13 = 54 %).

In addition to the MEDAS, participants were asked to complete a 33-page long semi-quantitative FFQ established and validated by the European Prospective Investigation into Cancer and Nutrition (EPIC) consortium^(22)^. The EPIC-FFQ provides the daily intakes of food groups (e.g. fruits, vegetables, nuts) and nutrients (e.g. fats, carbohydrates, protein). Data from the EPIC-FFQ were adjusted for energy intake^(23)^. Since the EPIC-FFQ does not measure adherence to the Mediterranean diet, we calculated the Mediterranean Diet Score, a commonly used score established by Trichopoulou *et al.* ^(24)^.

Thus, in the present analyses, we included two independent scores that determine adherence to the Mediterranean diet, that is the MEDAS-Score and the Mediterranean Diet Score. Dietary data for all variables and all time points are shown in online Supplementary Table 1.

### Biological measurements

The methodology to assess faecal fatty acids using GC^(17)^ and plasma fatty acids using GC with MS^(12)^ has previously been described in detail. The proportion (%) of each fatty acid in the total fatty acid composition (=100 %) was determined, and each fatty acid is shown as the proportion of the respective fatty acid. Faecal fatty acids were assessed in 2020 at the Université catholique de Louvain (Belgium).

The barrier biomarkers plasma LBP and faecal zonulin were assessed using enzyme-linked immunosorbent assays following the manufacturer’s protocols (zonulin, REF K5600, Immundiagnostik AG; LBP, REFs DY870-05 and DY008, Bio-Techne GmbH).

### Statistical analyses

In this explorative analysis, we included all sixty-eight participants from the completed LIBRE-1 study. The main focus of the present analysis was to assess possible changes in the faecal fatty acid composition, especially in the proportion of faecal long-chain *n*-3 PUFA upon intervention. Considering the mean change in the summarised proportions of EPA, DPA and DHA in the faecal fatty acids between BL and month 3, a *post hoc* power calculation showed a power of 81 % given a two-sided significance level of 5 % (intervention group −0·2 % (sd 0·7 %) (mean (sd)); control group 0·2 % (sd 0·4 %)) (two-sided Wilcoxon–Mann–Whitney test; G * Power software, Heinrich Heine University Duesseldorf).

As both gut barrier biomarkers and most fatty acids were not normally distributed (Shapiro–Wilk test), non-parametric tests were used. Differences between the intervention and control groups were tested using Fisher’s exact test for categorical variables or Mann–Whitney *U* tests for quantitative data. Within-group differences over time were assessed using Wilcoxon matched pairs signed-rank tests. To assess changes over time, we calculated the shift for each parameter (BL values subtracted from the respective values at time points V1 and V2, shown as ΔV1-BL and ΔV2-BL). Correlations were determined using Spearman’s rank coefficient with shift values. A *P* < 0·08 was considered as a trend, and a *P* < 0·05 was considered as statistically significant.

As these analyses are not confirmatory but to generate hypotheses to be further examined in larger studies, for example, LIBRE-2, we did not perform *post hoc* adjustment for multiple testing. To compensate for random findings in the correlation analyses, we would only draw conclusions based on results that can be found (i) consistently in both study arms and/or (ii) for both shifts ΔV1-BL and ΔV2-BL. Also, we only included those parameters in the correlation analyses that significantly changed during the intervention. Statistical analyses were performed using GraphPad Prism version 9·1·0 (GraphPad Software). Data are shown as medians with interquartile ranges (25th; 75th percentiles).

## Results

### Baseline characteristics

At BL, the intervention and control groups had similar numbers of women with previously diagnosed breast cancer, vegetarians and smokers, and they did not differ in age, BMI (Table 1) and physical fitness (data not shown).

**Table 1.** Patient characteristics at baseline

Parameters	Intervention group		Control group		P*
	*n*	%	*n*	%	
	*n* 33		*n* 35		
Diseased^†^ (*n* (%))	23	69·7	23	65·7	0·80
Vegetarians (*n* (%))	2	6·1	4	11·4	0·67
Smokers (*n* (%))	4	12·1	4	11·4	0·99
	Median	25th; 75th percentiles	Median	25th; 75th percentiles	
Age (years)	42	35; 49	41	35; 50	0·84
BMI (kg/m^2^)	23	21; 28	24	21; 28	0·49

*Between-group difference. ^†^Previously diagnosed with breast cancer. Total numbers and percentages (diseased, vegetarians, smokers) or median and interquartile ranges (age, BMI) are shown. Statistics: Fisher’s exact test (categorical variables) and Mann–Whitney *U* test (numerical data).

Both groups showed similar adherence to the Mediterranean diet according to the Mediterranean Diet Score at BL, yet the MEDAS-Score was slightly higher in the intervention group compared with the control group (50 % *v*. 42 %, *P* = 0·045) (online Supplementary Table 1). Besides the small difference in the MEDAS-Score, there was no BL difference in diet. Faecal proportions of all faecal fatty acids assessed, as well as the levels of the intestinal barrier biomarkers LBP and zonulin, did not differ between the groups at BL (Table 2 and online Supplementary Table 1).

**Table 2.** Changes in the faecal fatty acid composition (%) during the study. Shown are the data for baseline (BL), for the shift between BL and month 3 (Δ V1-BL) and for the shift between BL and month 12 (Δ V2-BL)

	Intervention group (*n* 33)						Control group (*n* 35)							
	BL		Δ V1-BL		Δ V2-BL		BL		Δ V1-BL		Δ V2-BL		Intervention *v.* control groups (P)	
	Median	25th; 75th percentiles	Median	25th; 75th percentiles	Median	25th; 75th percentiles	Median	25th; 75th percentiles	Median	25th; 75th percentiles	Median	25th; 75th percentiles	Δ V1-BL	Δ V2-BL
Fatty acids (%)*														
16:0 (Palmitic acid)	19	14; 26	**–1·4**	–11; −0·7*	**–3·3**	–15; 2·0**	16	13; 25	**2·5**	–1·0; 5·2*	0·6	–3·1; 4·6	**< 0·001**	**0·02**
16:1	0·2	0·1; 0·3	**–0·1**	–0·1; 0·0**	0·1	–0·2; 0·1	0·2	0·1; 0·3	**0·1**	**0·1; 0·2** ^**(**^ * ^**)**^	0·0	–0·1; 0·1	**< 0·001**	0·56
16:1 (Palmitoleic acid, *n*-7)	0·6	0·4; 0·9	**–0·2**	–0·5; 0·0**	**–0·1**	**–0·4; 0·1** ^**(**^ * ^**)**^	0·4	0·3; 0·6	0·1	–0·1; 0·3	0·0	–0·1; 0·1	**< 0·001**	**0·03**
18:0 (Stearic acid)	15·	8·1; 23	–0·9	–6·3; 2·3	**–5·9**	–12; 0·5*	11	7·9; 22	**2·6**	–0·7; 9·1*	1·5	–2·5; 4·8	**0·01**	**0·01**
18:1 (Elaidic acid)	0·3	0·2; 0·5	0·1	–0·1; 0·4	0·1	–0·2; 0·3	0·3	0·2; 0·6	0·0	–0·2; 0·3	0·0	–0·3; 0·1	0·64	0·64
18:1 (Vaccenic acid)	3·0	2·2; 5·9	**0·9**	**–1·5; 4·3** ^**(**^ * ^**)**^	–0·5	–3·0; 2·7	4·0	2·2; 7·9	0·2	–1·5; 2·4	–0·3	–2·4; 3·6	0·42	0·66
18:1 (Oleic acid, *n*-9)	25	19; 37	–1·4	–13; 11	3·4	–5·9; 15·8	27	18; 36	–1·8	–9·8; 4·8	3·1·	–10; 11·8	0·53	0·35
18:1^C11^	1·3	1·1; 1·7	–0·2	–0·5; 0·3	0·1	–0·5; 0·4	1·2	1·1; 1·5	0·0	–0·3; 0·3	0·1	–0·3; 0·4	0·43	0·62
18:2 (Linoleic acid, *n*-6)	16	11; 25	**5·9·**	**–6·7; 15** ^**(**^ * ^**)**^	**8·7**	–2·4; 17*	20	11; 32	**–4·2**	–15; 2·5*	–0·3·	–13; 4·8	**0·01**	**< 0·001**
18:2 (Rumenic acid)	0·6	0·2; 1·0	0·3	–0·2; 0·8	0·1	–0·6; 0·8	0·6	0·3; 1·0	0·1	–0·4; 0·7	0·1	–0·5; 0·5	0·77	0·9
18:2^T10C12^	0·1	0·1; 0·1	0·1	–0·1; 0·1	0·1	–0·1; 0·1	0·1	0·0; 0·1	0·0	–0·1; 0·1	0·0	–0·1; 0·1	0·19	0·92
18:2^C9C11^	0·1	0·1; 0·2	0·1	0·1; 0·2	0·1	–0·1; 0·1	0·1	0·0; 0·1	0·0	–0·1; 0·1	0·0	–0·1; 0·1	0·39	0·52
18:2^T11T13^	0·1	0·1; 0·1	–0·1	–0·1; 0·1	**–0·1**	**–0·1; 0·0** ^**(**^ * ^**)**^	0·1	0·0; 0·1	**0·1**	**0·0; 0·1** ^**(**^ * ^**)**^	**0·1**	0·0; 0·1*	**0·02**	**0·01**
18:2^T9T11^	0·1	0·1; 0·2	0·1	0·0; 0·1	0·1	–0·1; 0·1	0·1	0·1; 0·1	0·1	0·0; 0·1	0·0	0·0; 0·1	0·08	0·79
18:3 (ALA, *n*-3)	1·7	0·7; 6·6	**1·5**	–4·0; 4·7*	**1·1**	–4; 3·5*	1·7	0·7; 5·7	0·1	–3·5; 5·9	0·3	–1·6; 7·5	0·64	0·44
18:3^C8T10C12^	0·4	0·2; 0·6	**–0·1**	–0·3; 0·0**	–0·1	–0·2; 0·1	0·3	0·2; 0·5	0·1	–0·2; 0·3	0·0	–0·2; 0·1	**0·01**	0·65
18:3^C9T11C13^	0·2	0·1; 0·3	**–0·1**	–0·1; 0·0***	**–0·1**	–0·1; 0·0*	0·1	0·1; 0·2	**0·1**	**0·0; 0·2** ^**(**^ * ^**)**^	0·0	–0·1; 0·1	**< 0·001**	**0·05**
18:3^T8T10C12^	0·1	0·1; 0·2	0·0	0·0; 0·1	0·0	0·0; 0·1	0·0	0·0; 0·1	0·0	0·0; 0·1	0·0	0·0; 0·1	0·89	1
18:3^T9T11T13^	0·1	0·0; 0·1	0·0	–0·1; 0·0	0·0	–0·1; 0·0	0·1	0·0; 0·1	0·0	–0·1; 0·0	0·0	–0·1; 0·0	0·76	0·56
20:0 (Arachidic acid)	0·8	0·5; 1·0	**–0·2**	–0·3; 0·1*	**–0·1**	–0·5; 0·1*	0·6	0·5; 0·9	**0·2**	–0·2; 0·5*	0·0	–0·2; 0·2	**< 0·001**	0·09
22:0 (Behenic acid)	0·6	0·3; 0·8	–0·1	–0·3; 0·1	–0·1	–0·4; 0·2	0·6	0·4; 0·8	**0·1**	–0·1; 0·5*	–0·1	–0·3; 0·2	**0·01**	0·85
20:4 (ARA, *n*-6)	0·2	0·1; 0·4	**–0·1**	–0·2; 0·0*	**–0·1**	–0·3; 0·0*	0·2	0·1; 0·2	0·0	0·0; 0·2	0·0	0·0; 0·1	**< 0·001**	**< 0·001**
20:5 (EPA, *n*-3)	0·1	0·1; 0·2	–0·1	–0·1; 0·0	–0·1	–0·1; 0·0	0·1	0·0; 0·1	0·0	–0·1; 0·1	0·0	–0·1; 0·1	0·3	0·44
22:5 (DPA, *n*-3)	0·2	0·1; 0·4	**–0·1**	–0·2; 0·0***	**0·1**	**–0·3; 0·0** ^**(**^ * ^**)**^	0·2	0·1; 0·3	0·0	–0·1; 0·2	0·0	–0·1; 0·1	**< 0·001**	0·15
22:6 (DHA, *n*-3)	0·2	0·1; 0·4	–0·1	–0·2; 0·0	–0·1	–0·2; 0·1	0·2	0·1; 0·2	0·1	–0·1; 0·2	0·1	0·0; 0·1	0·11	**0·04**

Median and interquartile ranges (25th; 75th percentiles) are shown. Difference between the study groups at baseline and difference between the group’s shifts were tested using the Mann–Whitney *U* test. There was no between-group difference at baseline. Within-group difference between baseline and V1/V2 was tested using the Wilcoxon signed-rank test (^(^*^)^ *P* < 0·08; *P < 0·05; **P < 0·01; ***P < 0·001; results with a *P* < 0·08 are shown in boldface). Abbreviations: ALA, *α*-linoleic acid; ARA, arachidonic acid; DPA, docosapentaenoic acid. *Numbers denote double bond positions, whereas C and T denote *cis*- and *trans*-configuration, respectively.

### Effect of the intervention on dietary habits and biomarkers of intestinal barrier integrity

As described in detail before^(12)^, adherence to the Mediterranean diet increased markedly in the intervention group for at least 1 year (ΔV1-BL and ΔV2-BL, all *P* < 0·01) (online Supplementary Table 1). In the control group, there was a mild but significant increase in the adherence to the Mediterranean between BL and V1 (all *P* < 0·01), which was absent at V2.

Participants from the intervention group increased the intake of the typically Mediterranean food groups, especially the intake of nuts, fish and seafood, vegetables, legumes, fruits, olives and vegetable oil (ΔV1-BL and ΔV2-BL, all *P* < 0·05). At the same time, the intake of processed meat (*P* < 0·05 for ΔV1-BL and ΔV2-BL) and by trend red meat (*P* = 0·07 for ΔV1-BL) decreased in the intervention group, but not in the control group.

Both gut barrier biomarkers decreased markedly in the intervention group (LBP and zonulin *P* < 0·01 for ΔV1-BL and ΔV2-BL), suggesting improved intestinal barrier integrity. In the control group, both biomarkers decreased in the first 3 months but returned to BL levels after 1 year (*P* < 0·05 for ΔV1-BL; *P* > 0·08 for ΔV2-BL).

Diet data and data for the intestinal barrier biomarkers for all time points are shown in detail in online Supplementary Table 1.

### Effect of the Mediterranean diet on the faecal fatty acid composition

**Fig. 1.**
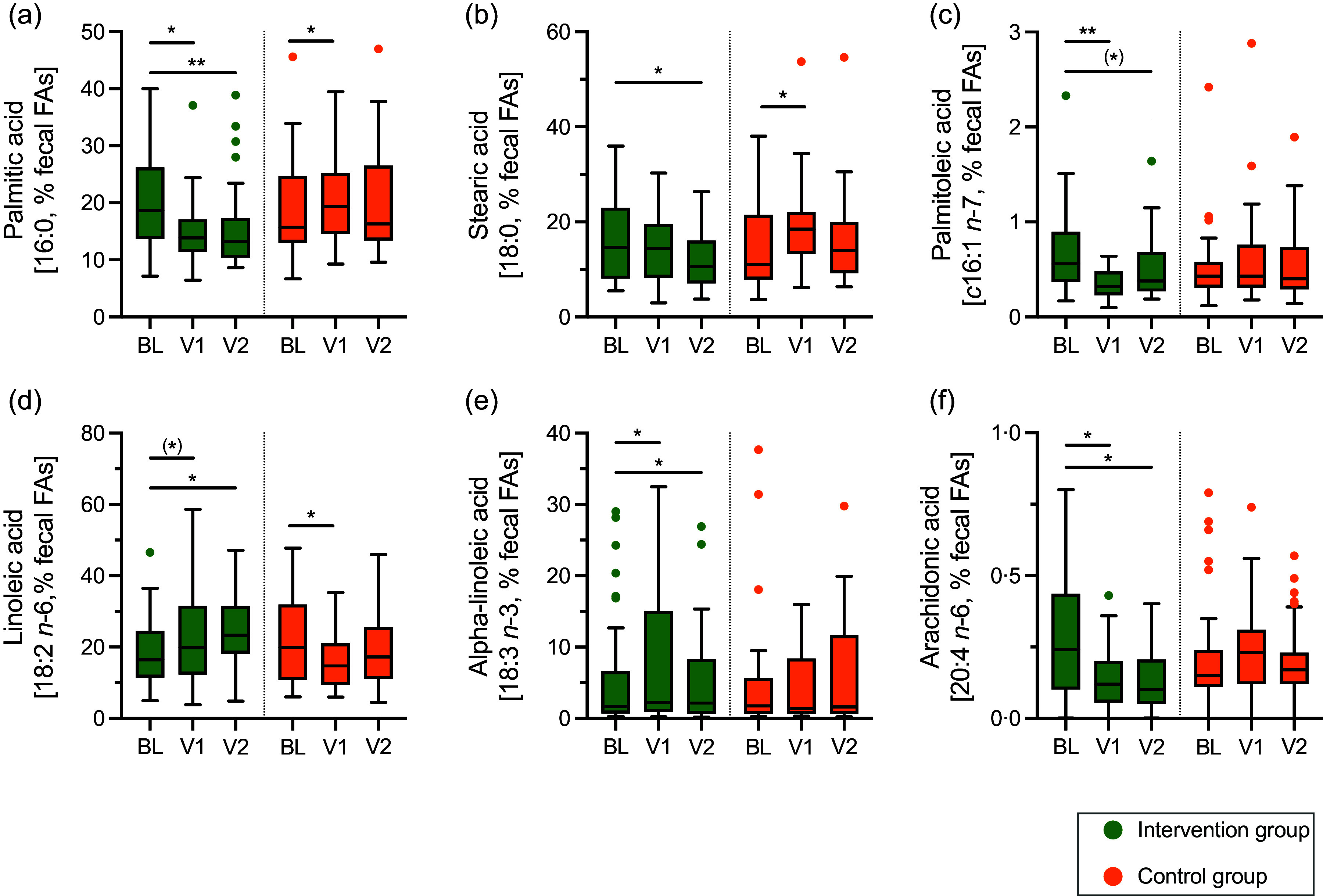
Effect of the intervention on the faecal fatty acid composition (a)–(f). Shown are data for baseline (BL), as well as after month 3 (V1) and month 12 (V2) for the proportion (%) of faecal fatty acids in the total faecal fatty acid composition (faecal FA). Tukey boxplots with median, whiskers (1·5 × interquartile ranges) and outliers are shown in green (intervention group; *n 33*) and orange (control group; *n 35*). Within-group difference to BL is indicated by asterisks (^(^*^)^ *P* < 0·08; *P < 0·05; **P < 0·01; Wilcoxon signed-rank test). This figure summarises data shown in Table 2.

We observed several changes in the faecal fatty acid composition in both study arms. Interestingly, several fatty acids that changed during the study showed different directions in the two study arms. For example, the proportions of the saturated fatty acids palmitic acid (16:0, Fig. 1(a)), stearic acid (18:0, Fig. 1(b)) and arachidic acid (20:0) decreased in the intervention group, while the proportions increased in the control group (Table 2). Also, the faecal proportion of the *n*-6 PUFA linoleic acid (18:2) increased in the intervention group and decreased in the control group (Fig. 1(d)) (Table 2).

In the intervention group, but not the control group, there was a decrease in the proportion of the *cis n*-7 palmitoleic acid (16:1, Fig. 1(c), Table 2) and the proportion of the *n*-6 PUFA arachidonic acid (20:4, Fig. 1(f), Table 2). Also, the proportion of the *n*-3 PUFA *α*-linoleic acid (18:3) increased solely in the intervention group (Fig. 1(e), Table 2). There was no effect of the intervention on faecal proportions of the *n*-3 PUFA EPA (20:5, *n*-3) and DHA (22:6, *n*-3) (Table 2).

Interestingly, there was a trend towards an increase in the faecal proportion of *trans* vaccenic acid (*t*18:1) in the intervention group (ΔV1-BL, *P* < 0·08), a monounsaturated fatty derived from microbial metabolism of linoleic acids^(17)^.

### The faecal proportion of palmitoleic acid is associated with adherence to the Mediterranean diet and the gut barrier biomarker lipopolysaccharide-binding protein

To test for possible associations, we ran correlation analyses including dietary parameters, the proportion of faecal fatty acids and the levels of the intestinal barrier biomarkers. For the correlation analyses, we used the shift values (ΔV1-BL and ΔV2-BL) and included only parameters that were significantly altered during the study (see Table 2 and online Supplementary Table 1).

**Fig. 2.**
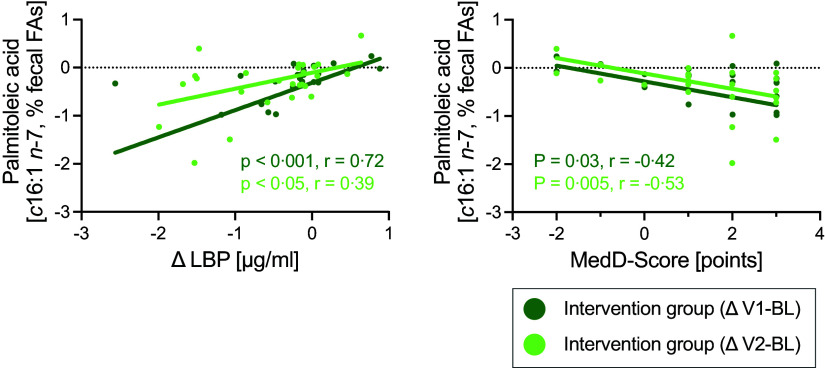
Adherence to the Mediterranean diet as well as plasma levels of lipopolysaccharide-binding protein (LBP) is associated with the faecal proportion of the *n*-7 monounsaturated palmitoleic acid. Shown are the correlations between the proportion (%) of palmitoleic acid in the total faecal fatty acid composition (faecal FA) and the adherence to the Mediterranean diet (assessed by the Mediterranean Diet Score (MedD-Score)) (a) and the proportion of palmitoleic acid and the plasma levels of the gut permeability biomarker LBP (b). Spearman correlations were conducted using shift values (baseline (BL) values subtracted from the respective values after month 3 (V1) and month 12 (V2). This figure summarises the findings shown in Table 3.

In the intervention group, the decrease in the faecal proportion of the *n*-7 palmitoleic acid (*c*16:1) correlated with the decrease in the levels of plasma LBP (Fig. 2(a)) and correlated inversely with adherence to the Mediterranean diet, assessed by the Mediterranean Diet Score (Fig. 2(b)) for both ΔV1-BL and ΔV2-BL (Table 3).

**Table 3.** Correlation analyses between the shifts in diet, the proportion of faecal fatty acids (FA) and intestinal barrier biomarkers. Shown are the data for the shift between baseline (BL) and month 3 (Δ V1-BL) and for the shift between BL and month 12 (Δ V2-BL). Correlations were only performed with parameters, which changed during the study (see Table 2 and online Supplementary Table 1)

1. Variable Δ	2. Variable Δ	Intervention group					
		Δ V1-BL			Δ V2-BL		
		*r*	R^2^	*P*	*r*	R^2^	*P*
16:1^C9^ (Palmitoleic acid) (% total faecal FA)	MedD-Score (Points)	–0·42 *n* 26	0·18	*P* = 0·03	–0·53 *n* 26	0·28	*P* = 0·005
16:1^C9^ (Palmitoleic acid) (% total faecal FA)	LBP (µg/ml)	0·72 *n* 25	0·52	*P* < 0·001	0·39 *n* 27	0·15	*P* < 0·05

Statistics: Spearman correlation. Abbreviations: LBP, lipopolysaccharide-binding protein; MedD-Score, Mediterranean Diet Score. We only show correlations that were found (i) consistently for the intervention group and the control group and/or (ii) consistently for the two shifts ΔV1-BL and ΔV2-BL. There were no significant results in the control group for Δ V1-BL and Δ V2-BL.

### The composition of faecal fatty acids is not associated with the composition of plasma fatty acids

Lastly, to assess possible associations between the compositions of faecal fatty acids and plasma fatty acids, we performed correlation analyses. In the analyses, we included the shift data (ΔV1-BL and ΔV2-BL) as well as the data for each time point (BL, V1, V2). We did not find any correlations between the proportion of faecal fatty acids and the proportion of plasma fatty acids that fit our selection criteria (correlation found in both groups and/or correlation found for both shifts and/or correlation found at two or more time points) (data not shown).

### Effect of the Mediterranean diet on the total amount of faecal fatty acids

As recommended^(25)^, we report both the absolute and relative data of the faecal fatty acid composition. As shown in detail in online Supplementary Table 2, the total amount of several faecal acids changed during the intervention. Notably, the total amount of faecal fatty acids increased in the intervention group, but not in the control group.

## Discussion

In the present study, we show that adherence to the Mediterranean diet induces several changes in the proportions of faecal fatty acids, especially in saturated fatty acids, but also in particular *n*-3 and *n*-6 PUFA. Interestingly, even though we have recently confirmed findings from cell lines^(26,27)^ and animals^(28–30)^ that diet-derived *n*-3 PUFA, especially DHA, are associated with an improvement in biomarkers of intestinal barrier integrity^(12)^, we did not find an association between faecal *n*-3 PUFA and biomarkers of intestinal barrier integrity in the present analyses.

### Adherence to the Mediterranean diet changed the faecal fatty acid composition

Adherence to the Mediterranean diet induced several changes in the faecal proportions of LCFA. Of interest, we did not see changes in the faecal proportions of the *n*-3 PUFA, DHA and EPA, commonly found in fish, even though the intake of fish increased in the intervention group. This finding is in line with data showing that after consuming different foods containing microencapsulated fish oil powder, only small amounts of *n*-3 PUFA are present in ileal effluent^(31)^. Our study now confirms these findings, showing that the increased intake of fish and seafood observed in the LIBRE cohort did neither increase the faecal proportion of these *n*-3 PUFA nor their total faecal amount. We assume that this finding is due to a very efficient intestinal absorption of *n*-3 PUFA^(32)^.

We observed a decrease in the faecal proportion of the *n*-6 PUFA arachidonic acid (20:4). There is increasing evidence that eicosanoids derived from arachidonic acid have abilities to regulate the intestinal epithelial barrier, yet the underlying mechanisms and the physiological relevance are not sufficiently understood^(33)^. Our data now indicate that the faecal proportion of arachidonic acid is not associated with impaired intestinal barrier integrity in humans. Though we base this finding on results from two validated intestinal barrier biomarkers, we suggest that future research in this field should assess intestinal barrier integrity by additional assessments, like the lactulose-mannitol-sucralose test.

We found a transient increase in the proportion of *trans* vaccenic acid (*t*18:1) in the intervention group in the first 3 months. This fatty acid, derived from microbial metabolism of linoleic acids, has previously been associated with the intake of specific dietary fibres, namely, chitin-glucan, a branched β-1,3/1,6 glucan^(17)^. The findings from this previous study are in line with the present results, showing that the faecal proportion of *trans* vaccenic acid was also increasing in the intervention group in the first 3 months. Affirming this, we found an inverse correlation between the change in the faecal proportion of *trans* vaccenic acid and the *n*-3 PUFA alpha-linoleic acid (18:3) in the first 3 months (*r* = –0·44, *P* = 0·01, Spearman correlation).

In the present study, we focused mainly on the faecal fatty acid composition expressed in percentage. This is due to the fact that fatty acid data expressed in percentage tends to exhibit lower inter-individual and intra-individual variability than absolute concentration and tends to be distributed normally, thereby increasing statistical power, especially when the total amount of fatty acids changes during the study course^(25)^. Furthermore, we chose to analyse our data using the fatty acid composition expressed in percentage to keep our results comparable with previous clinical trials investigating the effect of dietary fibres on faecal fatty acid patterns, which were expressed in percentage^(16,17)^.

### Faecal *n*-3 fatty acids are not associated with biomarkers of intestinal barrier integrity

Previously, *in vitro* studies^(26,34,35)^ and studies in rodents^(36–38)^ showed that *n*-3 PUFA affect the tight junction proteins occludin and zonula occludens-1, which are essential for effective cell–cell connections, preventing uncontrolled paracellular permeability of, for example, lipopolysaccharides into the bloodstream, increasing the production of LBP^(3)^. Furthermore, it was shown that *n*-3 PUFA induce the G protein-coupled receptor 120, which exerts anti-inflammatory effects and increases tight junction stability^(39–41)^, potentially decreasing uncontrolled paracellular permeability.

In contrast to plasma proportions^(12)^, the faecal proportion of *n*-3 PUFA was not associated with biomarkers of intestinal barrier integrity in the LIBRE cohort. We assume that this is due to the fact that *n*-3 PUFA are absorbed throughout the passage via the digestive tract^(32)^. Possibly, there might be beneficial effects of luminal *n*-3 PUFA on intestinal barrier integrity in proximal parts of the intestinal tract. However, assessment of these fatty acids in faecal samples does not allow to assess this, as most of the *n*-3 PUFA get absorbed along the digestive tract. Based on our findings, we propose that future studies should investigate the association between faecal fatty acids and intestinal barrier integrity using samples taken directly from the small intestine or proximal parts of the large intestine, for example, by capsules^(42)^.

It has been recently shown *in vitro* that long-chain saturated fatty acids (esp. 16:0 and 18:0) increase intestinal permeability, presumably by inducing severe mitochondrial alterations in enterocytes, marked by a diminution of adenosine triphosphate production due to high proton leak, oxidative phosphorylation uncoupling, mitochondrial network remodelling and reactive oxygen species generation^(43)^. Despite the finding that the faecal proportion of the two long-chain saturated fatty acids 16:0 and 18:0 decreased within the first 3 months in the present clinical study, there was no association between their faecal proportion and biomarkers of barrier function. This is in contrast to our previous findings, where we showed an association between the plasma proportion of long-chain saturated fatty acids and biomarkers of intestinal barrier integrity^(12)^. The reason for this difference is currently unknown.

### Faecal palmitoleic acid is associated with adherence to the Mediterranean diet and biomarkers of intestinal barrier integrity

We observed a decrease in the faecal proportion of the *n*-7 monounsaturated *cis*-palmitoleic acid (*c*16:1). In humans, this fatty acid mainly originates from *de novo* lipogenesis mediated by stearoyl-CoA desaturase-1, the rate-limiting enzyme catalysing the synthesis of monounsaturated fatty acids from saturated fatty acids. Consistent with findings from others^(44,45)^, we found an inverse association between palmitoleic acid and adherence to the Mediterranean diet. The role of palmitoleic acid in health and disease is not fully understood and is being discussed controversially, especially because previous results from studies in animals and humans partly showed opposing metabolic effects, as summarised by Frigolet and Gutiérrez-Aguilar^(46)^. In murine models, palmitoleic acid could effectively repair the intestinal mucosal barrier^(47)^. In this study, the authors stated a potential role of the gut microbiota, declaring that palmitoleic acid rewired disrupted intestinal barrier and reprogrammed gut microbiota by selectively increasing the abundance of anti-inflammatory gut bacteria such as *Akkermansia muciniphila* ^(47)^.

A study in high-fat diet-fed mice also showed that palmitoleic acid promotes intestinal tight junction integrity^(48)^. In detail, palmitoleic acid significantly increased the level of occludin and claudin-1, two proteins that regulate the formation, maintenance and function of tight junction in the intestine^(48)^. Furthermore, previous studies on metabolically active tissues have revealed that palmitoleic acid suppresses inflammatory cytokine production and attenuates inflammation^(49,50)^.

In contrast to these preclinical findings, our data indicate that a higher faecal proportion of palmitoleic acid is associated with higher levels of the gut barrier biomarker LBP, implying higher intestinal permeability. Here, further clinical studies are needed to investigate the association between this fatty acid and intestinal barrier integrity. These future studies should assess whether the opposing effects between preclinical studies (cell line, tissue and animal studies) and clinical studies described by Frigolet and Gutiérrez-Aguilar^(46)^ are also found in terms of intestinal barrier integrity in humans.

### Limitations and strengths

A limitation of our study is that we only included women with BRCA mutations with associated mild intestinal barrier dysfunction. To what extent our finding will also hold true for other populations with more pronounced intestinal barrier dysfunction needs to be explored in future studies. In a validation study, both intestinal barrier biomarkers used in the present study were validated in healthy individuals without pathogenic BRCA germline mutations^(5)^. Also, faecal zonulin was validated as a well-suited barrier biomarker for overweight and obese individuals, but not for normal-weight individuals without barrier dysfunction^(5)^. These points need to be regarded when interpreting the current findings, which are derived from normal-weight women with impaired barrier function due to pathogenic BRCA germline mutations. Also, the approach of the present study is of an explorative nature, investigating outcome parameters that were not planned initially. The reasons why we still assessed these parameters are new developments in the field, which were not known at the time the LIBRE study was planned in 2013. For example, gut barrier biomarkers were validated for clinical use in 2021^(5)^, and larger studies investigating the effect of dietary fibre on faecal LCFA are from 2020 and 2021^(16,17)^. A strength of our study is the study design of a randomised controlled trial and a rigorous approach in the statistical analyses. To omit reporting random findings, we only show correlation results that were found consistently in the two study groups and/or found for more than one time point.

### Conclusion

To the best of our knowledge, this is the first clinical study investigating a potential association between faecal LCFA and biomarkers of intestinal barrier integrity. Our findings show that there is only a small association between faecal LCFA and biomarkers of intestinal barrier integrity, in contrast to what was previously shown for plasma fatty acids^(12)^.

In conclusion, our data show that adherence to the Mediterranean diet induces distinct changes in the faecal fatty acid composition. We showed that the faecal proportion of palmitoleic acid, a *n*-7 monounsaturated fatty acid, was associated with the intestinal barrier biomarker LBP. In contrast to *n*-3 PUFA assessed in plasma, we found no association between faecal *n*-3 PUFA and biomarkers of intestinal barrier integrity.

## Supporting information

Seethaler et al. supplementary material 1 Seethaler et al. supplementary material

Seethaler et al. supplementary material 2 Seethaler et al. supplementary material

## Supplementary material

For supplementary material/s referred to in this article, please visit https://doi.org/10.1017/S0007114524001788.
