# Supplementary material for: Effect of the Mediterranean diet on the faecal long-chain fatty acid composition and intestinal barrier integrity: an exploratory analysis of the randomised controlled LIBRE trial
Source: Br J Nutr. 2024 Oct 21;132(9):1152–60. doi: 10.1017/S0007114524001788 (PMC11617107; doi:10.1017/S0007114524001788)
Supplement: Seethaler et al. supplementary material 1 — Seethaler et al. supplementary material [file S0007114524001788sup001.pdf]

Effect of the Mediterranean diet on fecal long-chain fatty acids and intestinal barrier integrity: An exploratory analysis of the randomized controlled LIBRE trial

Benjamin Seethaler[1], Maryam Basrai[1], Audrey M. Neyrinck[2], Walter Vetter[3], Nathalie M. Delzenne[2], Marion Kiechle[4], Stephan C. Bischoff[1]

British Journal of Nutrition

[1]Institute of Nutritional Medicine, University of Hohenheim, Stuttgart, Germany

[2]Metabolism and Nutrition Research Group, Louvain Drug Research Institute, UCLouvain, Université catholique de Louvain, Brussels, Belgium

[3]Institute of Food Chemistry, University of Hohenheim, Stuttgart, Germany

[4]Department of Gynecology, Center for Hereditary Breast and Ovarian Cancer, Klinikum Rechts der Isar, Technical University Munich and Comprehensive Cancer Center Munich, Munich, Germany

Corresponding author: Stephan C. Bischoff, M.D., Professor of Medicine, Institute of Nutritional Medicine, University of Hohenheim, Fruwirthstr. 12, 70593 Stuttgart, Germany. Phone: +4971145924101. E-mail: bischoff.stephan@uni-hohenheim.de

Supplementary material

Supplementary Figure 1 Page 2

Supplementary Table 1 Pages 3,4

Supplementary Table 2 Page 5

Supplementary Figure 1

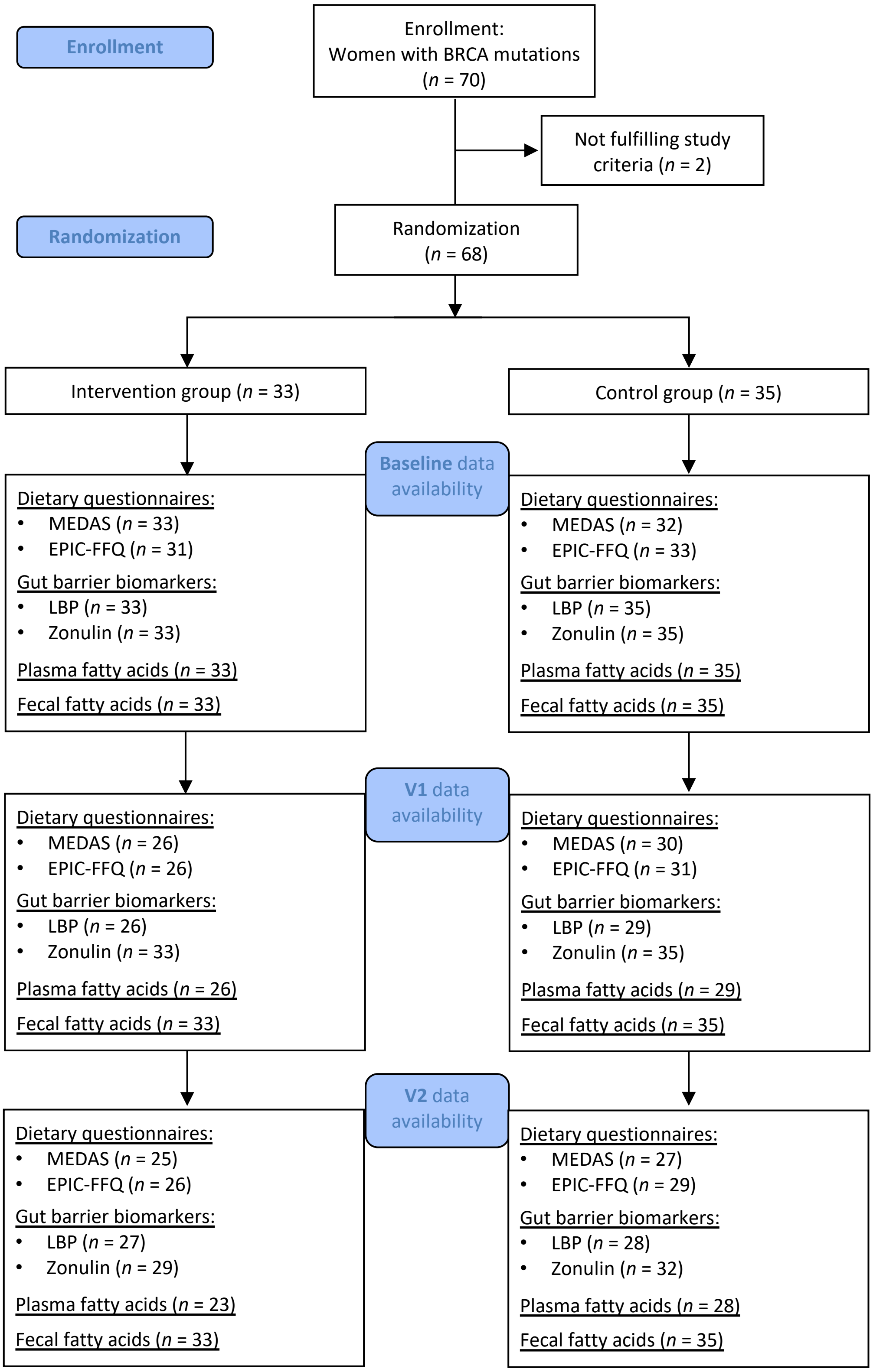

CONSORT flow diagram showing data availability for each study visit. Two women were excluded after enrolent due to unclear BRCA status. **Abbreviations:** BRCA, breast cancer gene; MEDAS, Mediterranean Diet Adherence Screener; EPIC-FFQ, European Prospective Investigation into Cancer and Nutrition – Food Frequency Questionnaire; LBP, lipopolysaccharide binding protein; V1, 3 months after baseline; V2, 12 months after baseline.

Supplementary Table 1. Changes in diet, BMI, and the barrier biomarkers plasma lipopolysaccharide binding protein (LBP) and fecal zonulin during the study. Shown are the adherence to the Mediterranean diet (MedD), the intake of nutrients and food groups according to the Food Frequency Questionnaire (FFQ), and the body mass index (BMI) for baseline (BL), for the shift between BL and month 3 (Δ V1-BL), and for the shift between BL and month 12 (Δ V2-BL).

	Intervention group			Control group			Intervention vs. control groups (P)	
	**BL (*n* = 31-33)**	**Δ V1-BL (*n* = 26)**	**Δ V2-BL (*n* = 22-26)**	**BL (*n* = 32-35)**	**Δ V1-BL (*n* = 30-31)**	**Δ V2-BL (*n* = 27-29)**	**Δ V1-BL**	**Δ V2-BL**
**MedD-scores**								
MEDAS [%]	50 (36;59)	**7.1 (0;29)****	**14 (0;21)****	42 (29;50)$^{\#}$	**7.1 (0;21.43)*****	0 (0;14)	0.55	0.22
MedD [points]	4 (4;5)	**1.5 (0.8;3)*****	**1 (-0.3;2.3)****	4 (3;6)	**1 (0;2)*****	0 (-1;2)	0.53	**0.08**
**Nutrients [g/d]**								
Plant protein	25 (22;31)	1.1 (-3.9;6.5)	2.7 (-0.7;7.5)$^{(*)}$	26 (21;32)	-0.7 (-3.2;3.1)	**-2.3 (-8.6;1.5)***	0.30	**0.003**
Animal protein	40 (31;50)	-0.9 (-9;6)	0.4 (-9;8)	45 (30;51)	-2.6 (-8;7)	**-5.2 (-13;-0.1)****	0.73	0.09
Cellulose	4.3 (3.7;5.1)	0.3 (-0.3;1.4)$^{(*)}$	**0.7 (-0.1;1.4)***	4.7 (3.8;5.6)	-0.3 (-1.1;0.6)	-0.4 (-1.2;0.4)$^{(*)}$	0.06	**0.002**
Lignin	1.3 (1;2)	0 (-0.3;0.2)	0.2 (-0.1;0.4)$^{(*)}$	1.4 (1.1;1.8)	**-0.1 (-0.4;0.1)***	-0.1 (-0.5;0.2)	0.16	0.05
Waterinsol. fibers	14 (11;18)	0.8 (-1.5;3.6)	**2.5 (-0.7;4.3)***	15 (12;18)	0.0 (-2.6;1.3)	**-1.4 (-3.6;0.8)***	0.05	**0.002**
Watersol. fibers	6.9 (5.6;8.6)	0.5 (-1;2.1)	**1.2 (0;2.7)***	7.0 (5.5;8.4)	0.0 (-1.5;0.8)	**-0.7 (-2.2;0.3)***	0.14	**0.001**
Polysaccharides	97 (80;130)	1.5 (-25;22)	5.0 (-8.4;39)	98 (87;137)	-1.0 (-23;8)	-13.3 (-29.3;12)$^{(*)}$	0.53	**0.02**
Non res. oligos.	0.3 (0.2;0.4)	**0.1 (0;0.2)***	0.1 (0;0.1)$^{(*)}$	0.3 (0.2;0.4)	0.0 (-0.1;0.1)	0.0 (-0.1;0.0)	**0.01**	**0.02**
Res. oligos.	1.2 (0.9;2.4)	0.0 (-0.1;0.7)	0.1 (-0.2;0.8)	1.1 (0.7;3.1)	0.0 (-0.2;0.3)	0.1 (-0.3;0.6)	0.43	0.51
Fibers	21 (17;26)	1.3 (-2.5;6.1)	**4.0 (-0.4;7.5)***	21 (18;26)	-0.3 (-4.0;2.0)	**-2.1 (-6.3;0.9)***	0.07	**0.001**
Fat	88 (75;102)	5.7 (-8.8;20)	5.2 (-2.4;11)	100 (85;115)	**-8.7 (-23;4)***	**-10.7 (-36;3)***	**0.04**	**0.001**
Carbohydrates	200 (160;248)	4.1 (-22;50)	17 (-18;34)	244 (188;276)	-9.7 (-40;15)$^{(*)}$	-19 (-77;16)$^{(*)}$	0.10	**0.02**
**Food groups [g/d]**								
Potatoes	34 (22;47)	-0.1 (-11;17)	0.1 (-4.2;2.9)	45 (34;67)	-0.6 (-19;11)	0.1 (-25.9;11.7)	0.42	1.00
Vegetables	131 (105;186)	**36 (-2.6;74)***	**26 (-14;69)***	133 (89;193)	-8.0 (-29.8;54)	-4.0 (-45;30)	0.10	0.07
Legumes	2.7 (1.7;4.8)	**1.8 (-0.6;8.9)****	**2.6 (-0.3;5.2)****	3.3 (1;4.9)	0.6 (-1.7;3)	0.4 (-2.3;2.5)	0.09	0.09
Fruits	176 (131;256)	**43 (-8;137)***	**55 (-1.3;119)****	211 (128;288)	-11 (-79.6;73)	12 (-59.8;69)	**0.03**	0.12
Nuts	2.8 (1;7.2)	**1.8 (-0.3;4.9)****	**2.4 (-0.3;6.2)***	1.5 (0.6;7.3)	0.2 (-0.7;1.7)	0.1 (-0.8;2.8)	0.05	0.09
Olives	0.2 (0.1;0.4)	0.1 (-0.2;0.3)	**0.2 (0;0.5)*****	0.2 (0;0.6)	0.0 (-0.1;0.3)	0 (-0.1;0.4)	0.81	0.11
Milk	125 (61;232)	11 (-16;71)	-2.1 (-62;50)	115 (52;214)	0.0 (-28;95)	-0.4 (-32;29)	0.78	0.55
Yoghurt	55 (19;88)	3.5 (-24;44)	-3.9 (-47;17)	41 (18;72)	0.0 (-21;18)	0.0 (-13;20)	0.31	0.23
Cereals	177 (142;250)	-5.1 (-56;53)	17 (-17;89)	176 (140;265)	-2.8 (-44;30)	-35 (-75;32)$^{(*)}$	0.91	**0.01**

Suppl. Table 1 (continued)	**Intervention group**			**Control group**			**Intervention vs. control groups (P)**	
	**BL (*n* = 31-33)**	**Δ V1-BL (*n* = 26)**	**Δ V2-BL (*n* = 22-26)**	**BL (*n* = 32-35)**	**Δ V1-BL (*n* = 30-31)**	**Δ V2-BL (*n* = 27-29)**	**Δ V1-BL**	**Δ V2-BL**
**Food groups [g/d]**								
Red meat	18 (8.7;27)	-2.6 (-14;0.8)(*)	-1.9 (-15;5.1)	20 (4.7;38)	-1.8 (-14;1.4)	-1.2 (-17;2.3)	0.65	0.81
Poultry	8.9 (2.8;20)	-0.6 (-12;3.1)	0.4 (-12;13)	10.6 (3.3;22)	-0.5 (-7.0;3.7)	-4.5 (-11;1)(*)	0.43	0.25
Processed meat	30 (19;52)	**-8.7 (-13;-0.3)****	**-4.3 (-15;3.5)***	31 (15;42)	-1.6 (-12;4.3)	0.9 (-9;6.1)	0.09	0.30
Fish	8.7 (8.6;22)	**11 (0;14)*****	**14 (0;22)*****	8.7 (1.9;22)	0.0 (0.0;7.0)	0.0 (-7.6;4)	0.07	**0.002**
Fish & seafood	12 (11;30)	**14 (-0.3;21)*****	**18 (0;26)*****	13 (8;30)	0.0 (-2.2;11)	0.0 (-9;4)	**0.04**	**0.002**
Egg	10 (7.1;16)	0.5 (-1.8;3.8)	**4.4 (-0.7;8.6*)**	10 (4.8;14)	-0.2 (-3.1;4.6)	0.9 (-2.5;4.8)	0.49	0.20
Vegetable oils	11 (6.9;16)	**4.4 (-1;15)***	**4.9 (-0.1;12)*****	9.0 (6.5;19)	0.5 (-2.8;7.0)	0.5 (-7.7;9)	0.13	**0.03**
Butter	6.6 (3.2;12)	-0.1 (-3.1;1.1)	0.5 (-2;6.1)	9.2 (2.6;14)	-0.1 (-3.5;3.3)	-0.2 (-5.2;3.7)	0.51	0.35
Confectionery	35 (21;59)	-2.1 (-18;4.9)	**-5.4 (-14;0.7)****	50 (28;65)	-5.6 (-18;7.2)	-4 (-22;7.2)	0.84	0.91
Wine	46 (7.5;90)	0.0 (-16;38)	3.5 (-4.2;55)	30 (10.8;108)	0.0 (-13;4.0)	0.0 (-17.3;67)	0.24	0.52
**BMI**								
BMI [kg/m^2]	23 (21;28)	-0.2 (-0.7;0.1)	0.1 (-0.6;0.7)	23 (21;28)	**0.32 (-0.1;0.81)***	0.27 (-0.48;0.72)	**0.002**	0.55
**Biomarker**								
LBP [µg/ml]	3.4 (3.1;3.8)	**-0.3 (-0.6;0.1)****	**-0.3 (-1.1;-0.1)*****	3.5 (3.1;4)	**-0.2 (-0.8;-0.1)*****	0.0 (-0.6;0.3)	0.21	**0.02**
Zonulin [ng/mg]	170 (132;328)	**-76 (-164;-12)****	**-74 (-197;15)****	180 (60;264)	**-59 (-186;15)***	-10 (-117;24)	0.58	0.18

Median and interquartile ranges (25th;75th percentiles) are shown. Difference between the study groups at baseline and difference between the group's shifts was tested using the Mann-Whitney *U* test (#difference between the groups at baseline; $p < 0.05$). Within group difference between baseline and V1/V2 was tested using the Wilcoxon signed-rank test ((*)P < 0.08; *P < 0.05; **P < 0.01; ***P < 0.001). Further abbreviations: MEDAS-Score, Mediterranean Diet Adherence Screener-Score; MedD-Score, Mediterranean Diet Score; oligos., oligosaccharides; watersol., watersoluble; waterins., waterinsoluble; res., resorbable.

Supplementary Table 2. Changes in fecal fatty acid composition (µg / mg feces) during the study. Shown are the data for baseline (BL), for the shift between BL and month 3 (∆ V1-BL), and for the shift between BL and month 12 (∆ V2-BL).

	Intervention group (n = 33)			Control group (n = 35)			Intervention vs. control groups (P)	
**Fatty acids [µg/mg feces][1]**	**BL**	**∆ V1-BL**	**∆ V2-BL**	**BL**	**∆ V1-BL**	**∆ V2-BL**	**∆ V1-BL**	**∆ V2-BL**
16:0 (Palmitic acid)	8.3 (5.3;11)	**1.2 (-1.3;5.9)***	**2.8 (-0.8;6.2)***	10 (7.3;11.7)	-0.8 (-2.9;2.4)	-1.0 (-2.7;4.8)	**0.04**	0.16
16:1^{T9}	0.1 (0.1;0.1)	0.1 (0.1;0.1)	0.1 (0.1;0.1)	0.1 (0.1;0.1)	0.1 (0.1;0.1)	0.1 (-0.1;0.1)	0.23	0.47
16:1 (Palmitoleic acid, *n*-7)	0.2 (0.1;0.4)	0.0 (-0.2;0.1)	0.1 (-0.1;0.3)$^{(*)}$	0.2 (0.2;0.3)	0.1 (-0.1;0.1)	0.0 (-0.1;0.1)	0.62	0.16
18:0 (Stearic acid)	5.6 (4.2;8.8)	**2.2 (-0.8;4.7)***	1.3 (-1.5;4.3)	7.0 (5.5;9.5)	0.1 (-1.5;3.2)	0.1 (-2.0;1.8)	0.20	0.25
18:1 (Elaidic acid)	0.1 (0.1;0.3)	**0.1 (0;0.3)****	**0.1 (0;0.3)***	0.2 (0.1;0.4)	0.0 (-0.2;0.1)	0.1 (-0.2;0.1)	**0.02**	**0.01**
18:1 (Vaccenic acid)	1.0 (0.6;2.6)	**0.7 (0;2.7)****	**0.6 (-0.4;2)$^{(*)}$**	**2.2 (1;4.1)$^{\#}$**	0.2 (-1.3;1.3)	-0.1 (-1.3;1.6)	0.06	0.21
18:1 (Oleic acid, *n*-9)	7.7 (4.5;26)	6.9 (-5.6;19.1)	**13.8 (-2.2;22)***	18 (6.5;27)	**-4.6 (-14.2;1.9)***	-0.8 (-13.3;12)	**0.01**	**0.01**
18:1^{C11}	0.5 (0.3;1.1)	**0.3 (-0.1;0.6)***	**0.3 (-0.1;1.1)****	0.8 (0.5;1)	-0.2 (-0.4;0.2)	0.1 (-0.2;0.3)	**0.01**	**0.02**
18:2 (Linoleic acid, *n*-6)	7.1 (3;13.6)	**4.4 (0.4;10)****	**5.4 (-1.4;29)****	11.2 (5;22)	**-6.4 (-13.1;1.4)****	-2.7 (-15;3.9)	**< 0.001**	**0.002**
18:2 (Rumenic acid)	0.2 (0.1;0.6)	**0.1 (0;0.9)****	**0.2 (-0.1;0.9)***	0.3 (0.2;0.6)	0.1 (-0.4;0.5)	0.0 (-0.3;0.3)	0.05	**0.02**
18:2^{T10C12}	0.1 (0.0;0.1)	0.1 (0.0;0.1)	**0.1 (0.1;0.2)***	0.1 (0.1;0.1)	0.0 (-0.1;0.1)	0.0 (-0.1;0.1)	0.11	0.08
18:2^{C9C11}	0.1 (0.1;0.1)	**0.1 (0.0;0.1)***	0.1 (0.0;0.1)	0.1 (0.1;0.1)	0.0 (-0.1;0.1)	0.0 (-0.1;0.1)	**0.04**	0.54
18:2^{T11T13}	0.1 (0.1;0.1)	0.1 (0.0;0.1)	0.1 (0.0;0.1)	0.1 (0.1;0.1)	**-0.1 (-0.1;0.1)***	0.0 (-0.1;0.1)	0.38	0.19
18:2^{T9T11}	0.1 (0.1;0.1)	**0.1 (0;0.1)****	**0.1 (0.0;0.1)****	0.1 (0.1;0.1)	0.1 (-0.1;0.2)	0.1 (-0.1;0.1)	0.11	**0.02**
18:3 (ALA, *n*-3)	0.4 (0.3;2.7)	**0.3 (-0.6;5.2)$^{(*)}$**	0.2 (-0.7;3.4)	0.7 (0.3;3.1)	0.1 (-1.5;1.8)	0.1 (-1.6;5.1)	0.19	0.64
18:3^{C8T10C12}	0.1 (0.1;0.2)	0.1 (0.0;0.1)	0.1 (0.0;0.1)	**0.2 (0.1;0.2)$^{\#}$**	0.0 (-0.1;0.1)	0.1 (0.0;0.1)	0.50	**0.01**
18:3^{C9T11C13}	0.1 (0.1;0.1)	0.1 (0.0;0.1)	0.1 (0.1;0.1)	0.1 (0.1;0.1)	0.1 (-0.1;0.2)	0.1 (-0.1;0.1)	0.83	0.99
18:3^{T8T10C12}	0.1 (0.1;0.1)	0.1 (0.0;0.1)	0.1 (0.1;0.1)	0.1 (0.1;0.1)	0.1 (0.0;0.1)	0.1 (-0.1;0.1)	0.39	0.10
18:3^{T9T11T13}	0.1 (0.1;0.1)	**0.1 (0.1;0.1)***	**0.1 (0.0;0.1)***	0.1 (0.1;0.1)	**0.1 (0.0;0.1)***	0.1 (-0.1;0.1)	**< 0.001**	0.08
20:0 (Arachidic acid)	0.3 (0.2;0.4)	**0.1 (0;0.2)***	0.1 (-0.1;0.2)	0.3 (0.3;0.4)	0.0 (-0.1;0.1)	0.0 (-0.1;0.1)	0.28	0.10
22:0 (Behenic acid)	0.2 (0.2;0.3)	**0.1 (0;0.2)****	**0.1 (-0.1;0.3)$^{(*)}$**	0.3 (0.2;0.4)	0.1 (-0.1;0.2)	0.0 (-0.2;0.1)	0.22	**0.01**
20:4 (ARA, *n*-6)	0.1 (0.1;0.1)	0.1 (0.0;0.1)	**-0.1 (-0.1;0.0)***	0.1 (0.1;0.1)	0.1 (-0.1;0.2)	0.1 (0.0;0.1)	0.35	0.09
20:5 (EPA, *n*-3)	0.1 (0.1;0.1)	0.0 (0.0;0.1)	0.1 (0.0;0.1)	0.1 (0.0;0.1)	**-0.01 (-0.1;0.2)$^{(*)}$**	0.1 (-0.1;0.1)	**0.04**	0.31
22:5 (DPA, *n*-3)	0.1 (0.1;0.1)	0.1 (0.0;0.1)	0.0 (0.0;0.1)	0.1 (0.1;0.1)	0.1 (0.0;0.1)	0.0 (-0.1;0.1)	0.39	0.74
22:6 (DHA, *n*-3)	0.1 (0.1;0.1)	0.0 (0.0;0.1)	0.1 (0.0;0.1)	0.1 (0.1;0.1)	0.0 (-0.1;0.1)	0.0 (-0.1;0.1)	0.43	0.82
Total fecal fatty acids	32.7 (24;76)	**26.8 (0.1;59)****	**24.9 (-10;72)***	55.5 (37;85)	-13.1 (-29;18)$^{(*)}$	-1.4 (-35;24)	**< 0.001**	**0.007**

Median and interquartile ranges (25th;75th percentiles) are shown. Difference between the study groups at baseline and difference between the group's shifts was tested using the Mann-Whitney *U* test (#p < 0.05). Within-group difference between baseline and V1/V2 was tested using the Wilcoxon signed-rank test ($^{(*)}$P < 0.08; *P < 0.05; **P < 0.01). Abbreviations: ALA, alpha-linoleic acid; ARA, arachidonic acid; EPA, eicosapentaenoic acid; DPA, docosapentaenoic acid; DHA, docosahexaenoic acid. [1]Numbers denote double bond positions whereas C and T denote *cis*- and *trans*-configuration, respectively.
